# PET-based immunomapping of intratumoral CD4^+^ cells to monitor acquired resistance to checkpoint inhibitors

**DOI:** 10.1126/sciadv.adw1924

**Published:** 2025-06-25

**Authors:** Stefania Pezzana, Simone Blaess, Bjoern Traenkle, Anna Schaefer, Lara Ruoff, Bredi Tako, Salvador Castaneda Vega, Philipp D. Kaiser, Teresa Wagner, Irene Gonzalez-Menendez, Leticia Quintanilla-Martinez, Alexander Rochwarger, Christian M. Schürch, Simon Riel, Martin Schaller, Evelien A. J. van Genugten, Iris A. E. van der Hoorn, Mark A. J. Gorris, Megan Steinvoort, Eva Peeters, I. Jolanda M. de Vries, Michel M. van den Heuvel, Erik H. J. G. Aarntzen, Andreas Maurer, Ulrich Rothbauer, Bernd J. Pichler, Manfred Kneilling, Dominik Sonanini

**Affiliations:** ^1^ Werner Siemens Imaging Center, Department of Preclinical Imaging and Radiopharmacy, University Hospital Tuebingen, University of Tuebingen, Tuebingen, Germany.; ^2^ NMI Natural and Medical Sciences Institute at the University of Tuebingen, Reutlingen, Germany.; ^3^ Department of Nuclear Medicine, University Hospital Tuebingen, University of Tuebingen, Tuebingen, Germany.; ^4^ Pharmaceutical Biotechnology, University of Tuebingen, Tuebingen, Germany.; ^5^ Cluster of Excellence iFIT (EXC2180) “Image-Guided and Functionally Instructed Tumor Therapies”, University of Tuebingen, Tuebingen, Germany.; ^6^ Department of Pathology and Neuropathology, University Hospital and Comprehensive Cancer Center Tuebingen, University of Tuebingen, Tuebingen, Germany.; ^7^ Department of Dermatology, University Hospital Tuebingen, University of Tuebingen, Tuebingen, Germany.; ^8^ Department of Medical Imaging, Radboud University Medical Center, Nijmegen, Netherlands.; ^9^ Department of Medical Biosciences, Radboud University Medical Center, Nijmegen, Netherlands.; ^10^ Department of Pulmonology, Radboud University Medical Center, Nijmegen, Netherlands.; ^11^ Department of Pulmonology, University Medical Center Utrecht, Utrecht, Netherlands.; ^12^ Department of Nuclear Medicine and Molecular Imaging, University Medical Center Groningen, Groningen, Netherlands.; ^13^ German Cancer Consortium (DKTK) and German Cancer Research Center (DKFZ), Partner site Tuebingen, Tuebingen, Germany.; ^14^ Department of Medical Oncology and Pneumology, University Hospital Tuebingen, University of Tuebingen, Tuebingen, Germany.

## Abstract

CD4^+^ T cells are crucial in shaping response and resistance to immunotherapy. To enhance our understanding of their multifaceted functions, we developed copper-64–radiolabeled nanobodies targeting the human CD4 receptor (^64^Cu-CD4-Nb1) for positron emission tomography (PET). In human CD4-receptor knock-in mice, ^64^Cu-CD4-Nb1 specifically accumulated in different orthotopic tumors, correlating with histological CD4^+^ cell densities. Based on intratumoral CD4^+^ cell distribution patterns within the core and periphery, we distinguished responders to combined αPD-1/4-1BB antibodies early on-treatment. CD4-PET identified resistance to αPD-1 monotherapy, which was mitigated by adding regulatory T cell–depleting α4-1BB antibodies. Patients with early-stage non–small cell lung cancer who relapsed after neoadjuvant αPD-L1 therapy revealed low CD4^+^ T cell densities in the tumor core. In human and mouse tumor tissues, regulatory T cells correlated with CD4^+^ cell densities. Thus, visualizing the spatial distribution patterns of CD4^+^ cells by PET offers mechanistic insights into CD4-mediated therapy efficacy, with great potential for guiding combinatorial immunotherapies in patients with cancer.

## Teaser

PET imaging of spatial CD4^+^ T cell distribution enables mechanistic insights into checkpoint inhibitor therapy.

## INTRODUCTION

T cells are crucially involved in the development and treatment of cancer. Whereas CD8^+^ cytotoxic T lymphocytes (CTLs) primarily mediate antitumoral immunity, CD4^+^ T cells are a heterogeneous group of cells with complex and multifaceted roles in either orchestrating or suppressing therapeutic immune responses (*1*, *2*). On the basis of their differentiation, CD4^+^ T helper cells can induce the cytotoxic activity of CTLs and activate B cells, dendritic cells, natural killer cells, and macrophages (*3*). In addition, specific subsets of CD4^+^ T cells exert direct antitumoral cytotoxicity and induce tumor senescence (*4*–*6*). In contrast, regulatory T (T_reg_) cells inhibit antitumoral immune responses within the tumor microenvironment (TME) (*7*).

T_reg_ cells have been identified as key drivers of resistance to immune checkpoint inhibitors (ICIs) targeting the programmed death protein 1 (PD-1) or its ligand (PD-L1) (*8*). PD-(L)1–directed ICIs have become the standard of care for many types of metastatic cancer (*9*, *10*), but overall response rates remain unsatisfactory, ranging from 12 to 40% depending on the tumor type (*11*–*15*). To improve therapeutic outcomes, αPD-(L)1 ICIs have been combined with other immunotherapies targeting additional immune checkpoints—such as cytotoxic T lymphocyte antigen 4 (CTLA-4), lymphocyte activation antigen 3 (LAG-3), chemokine receptor 7 (CCR-7), and 4-1BB—or with tumor vaccination. All of these approaches have been shown to specifically modulate CD4^+^ T cell function (*16*–*21*).

Positron emission tomography (PET), a noninvasive molecular imaging method, enables visualization of immune cells by using radiolabeled antibodies or antibody fragments targeting cell-specific surface markers (*22*). To better understand the mechanisms underlying CD4-mediated immunity and to individually stratify patients for combinatorial immunotherapies, various radiotracers, including full-length antibodies, radiolabeled F(ab′)_2_ fragments, engineered scFv dimers (cys-diabodies), or scFv-CH3 (minibodies), have been developed for the specific targeting of mouse, nonhuman primate, or human CD4^+^ cells (*23*–*29*). Recently, we compared zirconium-89 (^89^Zr)–labeled human and mouse CD4-specific minibodies in different syngeneic tumor models and successfully identified responders to combined αPD-L1/αLAG-3 ICI therapy in an MC38 tumor mouse model based on the whole-tumor PET uptake (*30*).

Increasing histology-driven evidence from mouse and human tumor tissues highlights the spatial location of specific CD4^+^ subsets within the TME and their proximity to other immune cells as critical biomarkers for determining ICI efficacy (*31*–*37*). To meet the requirement for highly specific and sensitive radiotracers capable of capturing the dynamic distribution patterns of immune cell with sufficient spatial resolution, we recently generated alpaca-derived single-domain antibodies [nanobodies (Nbs)] that target the human CD4 receptor for clinical PET imaging of CD4^+^ T cells in vivo (*38*). Because of their low molecular and picomolar binding affinities, Nbs offer advanced imaging properties including rapid tissue penetration and clearance from blood (*39*). From a set of 78 positive binders, we selected CD4-Nb1 as the lead candidate based on its binding characteristics, recognized epitopes, and lack of effects on T cell proliferation, activation, or cytokine release in vitro. CD4-Nb1 was subsequently radiolabeled with copper-64 (^64^Cu) for in vivo imaging (*38*).

In this study, we applied ^64^Cu-CD4-Nb1 to track intratumoral CD4^+^ T cells in human CD4 receptor knock-in (hCD4-KI) and wild-type (WT) C57BL/6J mouse models to explore whether clinically relevant changes CD4^+^ cells within the TME and lymphatic organs can be visualized by in vivo PET imaging. Spatially resolved PET uptake patterns observed during αPD-L1 monotherapy and combined ICI therapy were correlated with histological distribution of CD4*^+^* cells and therapy response. Notably, we demonstrate that differential core and marginal CD4^+^ cell quantifications of whole tumor tissues derived from patients with non–small cell lung cancer (NSCLC) after neoadjuvant αPD-L1 therapy were predictive of therapy resistance. These findings underscore the potential of ^64^Cu-CD4-Nb1 PET imaging to provide mechanistic insights into CD4-mediated immune responses and resistance, paving the way for its use in guiding combinatorial immunotherapy strategies in patients with cancer.

## RESULTS

### ^64^Cu-CD4-Nb1 specifically binds to human CD4 in vitro and in vivo

**Fig. 1.**
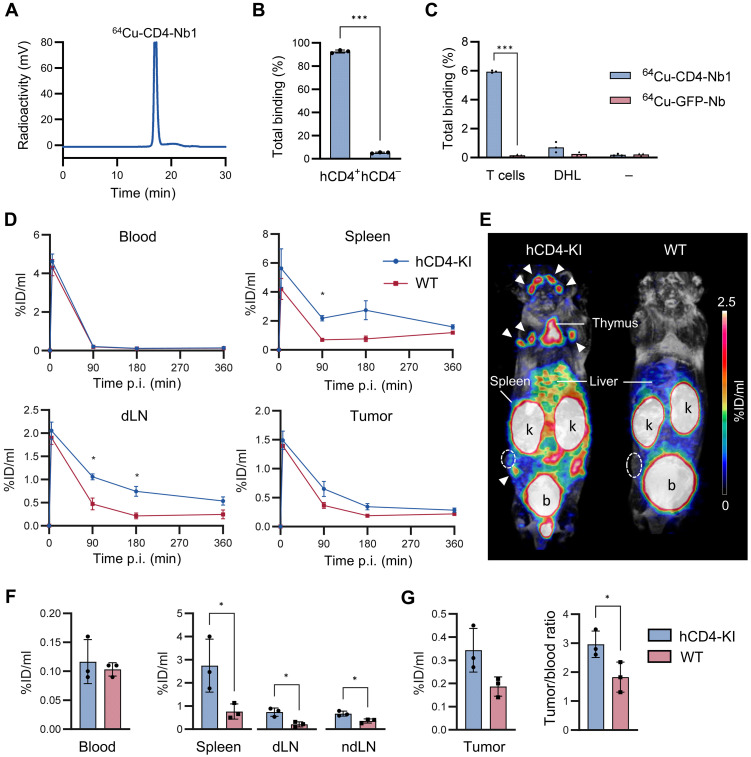
Specific binding and biodistribution dynamics of ^64^Cu-CD4-Nb1. (**A**) High-performance liquid chromatography chromatogram of ^64^Cu-CD4-Nb1. Representative data from >3 independent experiments. (**B**) Immunoreactive fraction of ^64^Cu-CD4-Nb1 using magnetic beads coated with human CD4 (hCD4^+^) or left uncoated (hCD4^−^). *n* = 3 per group, means ± SD, representative data from >3 independent experiments. (**C**) In vitro binding of ^64^Cu-CD4-Nb1 and a control Nb (^64^Cu-GFP-Nb) to freshly isolated hCD4^+^ T cells, hCD4^−^ DHL cells, or no cells (−). *n* = 3 per group. (**D**) ^64^Cu-CD4-Nb1 PET uptake dynamics 5 to 360 min post-tracer injection (p.i.) in the blood, spleen, draining lymph nodes (dLNs), and tumors (*n* = 3 per group, means ± SD). The data are presented as the mean percent of the injected dose per ml (%ID/ml). (**E**) Representative maximum intensity projection PET images overlaid with anatomical MR images acquired 180 min after ^64^Cu-CD4-Nb1 injection in human CD4 knock-in (hCD4-KI) or WT mice with orthotopic PyMT triple-negative breast cancer tumors. The tumors are outlined with white circles, and the lymph nodes are highlighted by white arrows; k: kidney; b: bladder. (**F**) ^64^Cu-CD4-Nb1 PET uptake quantification 180 min after tracer injection in the blood, spleen, dLNs, and nondraining lymph nodes (ndLNs). (**G**) ^64^Cu-CD4-Nb1 PET uptake quantification 180 min after tracer injection in PyMT tumors and the tumor-to-blood ratio. Pairwise comparisons were performed with Student’s *t* test and corrected for multiple comparisons using the Holm-Sidak method (*P < 0.05; ***P < 0.001).

For in vivo PET imaging of CD4^+^ cells, our CD4-Nb1 was conjugated site-specifically to p-NCS-benzyl-NODA-GA (NODAGA) and radiolabeled with ^64^Cu, with labeling efficiencies of over 90% (Fig. 1A). We revealed a maximum binding of 92.6% for ^64^Cu-CD4-Nb1 using hCD4-coated beads (Fig. 1B) and confirmed specific binding to freshly isolated hCD4^+^ T cells (Fig. 1C).

To determine the CD4-specific biodistribution kinetics in vivo, we analyzed ^64^Cu-CD4-Nb1 uptake in orthotopic S2WTP3 polyoma virus middle T antigen (PyMT) breast cancer–bearing hCD4 knock-in (hCD4-KI) and WT C57BL/6J mice (fig. S1A). Whereas ^64^Cu-CD4-Nb1 was rapidly cleared from the blood within the first 90 min after injection in both groups, we observed increased PET uptake in the lymphatic organs and PyMT tumors of hCD4-KI mice over time in vivo (1.5 to 6 hours) (Fig. 1D and fig. S1B) and ex vivo (6 hours) (fig. S1D). At the 3-hour imaging time point, CD4-derived PET signals could be clearly distinguished visually (Fig. 1E) and quantification revealed ~3.6-fold higher uptake in the spleen, ~3.5-fold higher uptake in tumor-draining lymph nodes, and ~1.8-fold higher uptake in PyMT tumors in hCD4-KI mice than in WT mice (Fig. 1, F and G, and fig. S1C). In contrast, organs with low CD4^+^ T cell infiltration, such as the lung, kidney, and muscle, as well as blood, showed a similar ^64^Cu-CD4-Nb1 uptake (fig. S1, B and C). This observation indicates that the presence or absence of the target antigen does not influence the biodistribution of the tracer in vivo.

### ^64^Cu-CD4-Nb1 PET imaging can detect clinically relevant changes in intratumoral CD4^+^ cell density

Next, we analyzed the sensitivity of ^64^Cu-CD4-Nb1 to detect variations in intratumoral CD4^+^ T cell densities. For this purpose, we orthotopically inoculated cells of the low-immunogenic B16F10 melanoma tumor cell line (*40*) and the immunogenic PyMT breast cancer cell line (*41*) into hCD4-KI or WT mice. The tumor-bearing mice were either left untreated or received combined αPD-1/α4-1BB ICI therapy for 7 days before ^64^Cu-CD4-Nb1 PET imaging (fig. S2A).

**Fig. 2.**
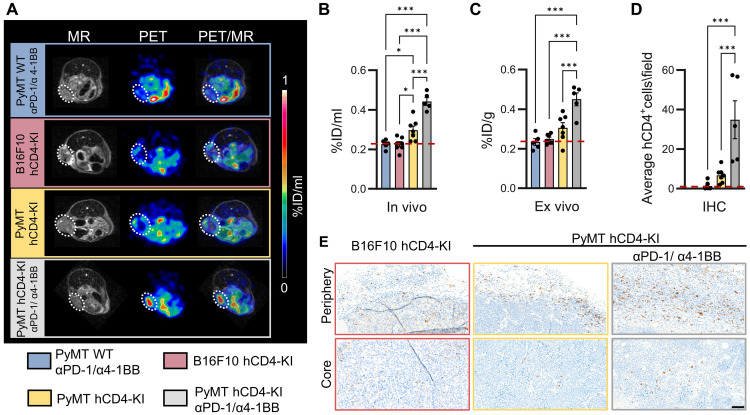
^64^Cu-hCD4-Nb1 PET to quantify varying intratumoral CD4^+^ cell densities. (**A**) Representative MR, PET, and coregistered PET/MR images 180 min after ^64^Cu-hCD4-Nb1 injection of PyMT and B16F10 tumors, which were implanted in WT (no hCD4 antigen) or hCD4-KI mice. Two groups were treated with αPD-1/α4-1BB antibodies for 1 week to increase CD4^+^ T cell infiltration. (**B**) Quantification of in vivo and (**C**) ex vivo ^64^Cu-CD4-Nb1 uptake at 180 min post-tracer injection and (**D**) mean hCD4^+^ cell/HPF ratio in the tumor center analyzed by IHC. Red horizontal lines represent background levels compared with those in the WT group. Pooled data from two independent experiments. *n* = 5 to 7 (two to four per experiment) per group, means ± SEM. (**E**) Representative hCD4 immunohistochemical images of the tumor core and periphery of PyMT and B16F10 tumors from hCD4-KI mice. Scale bar, 50 μm. Pairwise comparisons were performed with one-way ANOVA and corrected for multiple comparisons using the Holm-Sidak method (*P < 0.05; ***P < 0.001).

In untreated hCD4-KI mice, ^64^Cu-CD4-Nb1 uptake was slightly higher in PyMT tumors (0.30 ± 0.05 %ID/ml) than in B16F10 tumors (0.23 ± 0.02 %ID/ml) (Fig. 2, A and B). In the ICI-treated PyMT tumor-bearing hCD4-KI mice, the ^64^Cu-CD4-Nb1 uptake was significantly higher (0.44 ± 0.04 %ID/ml) than in the ICI-treated PyMT tumor-bearing WT mice. The latter showed a ^64^Cu-CD4-Nb1 uptake of 0.23 ± 0.02 %ID/ml, similar to that of the poorly immunogenic B16F10 melanomas (Fig. 2, A and B, and fig. S3A). Quantitative ex vivo biodistribution analysis of the tumors demonstrated values similar to the in vivo uptake values for each group (Fig. 2C and fig. S3C). The immunohistochemical analysis revealed that the CD4^+^ T cell densities correlated with ^64^Cu-CD4-Nb1 in vivo and ex vivo uptake when corrected for nonspecific background ^64^Cu-CD4-Nb1 uptake in tumors of WT animals (Fig. 2D). To note, we observed an accumulation of ^64^Cu-CD4-Nb1 preferentially at the tumor margins of nontreated PyMT tumors (Fig. 2A). In sharp contrast, in ICI-treated mice ^64^Cu-CD4-Nb1 mainly accumulated within the tumor cores (Fig. 2A). This ICI-related CD4^+^ T cell infiltration into the TME was also confirmed by immunohistochemical staining (Fig. 2, D and E).

**Fig. 3.**
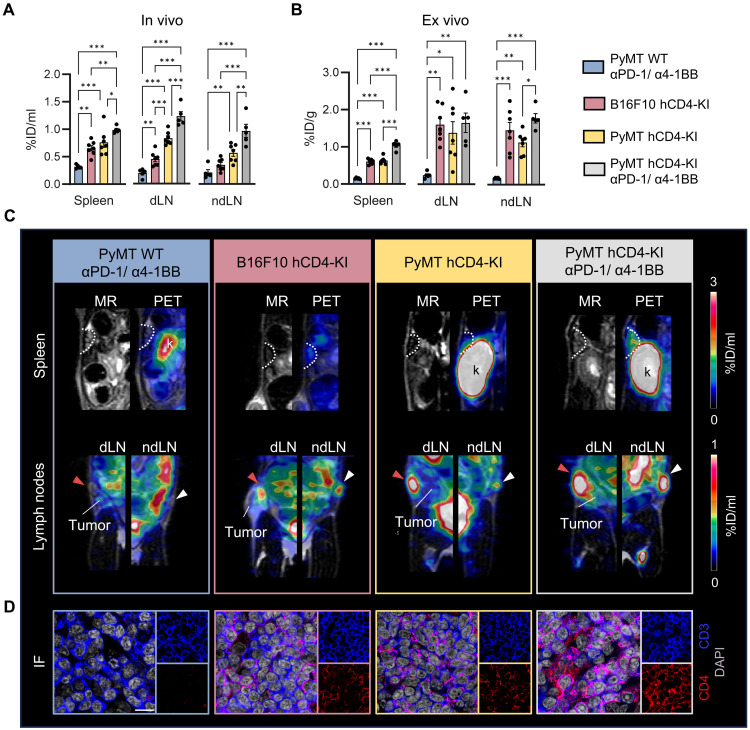
Noninvasive visualization of lymphatic organs in mice treated with or without αPD-1/α4-1BB immunotherapy. (**A**) In vivo and (**B**) ex vivo quantification of the spleen, dLNs, and contralateral ndLNs of PyMT and B16F10 tumor-bearing hCD4-KI and WT mice acquired, with or without αPD-1/α4-1BB treatment, 180 min after ^64^Cu-CD4-Nb1 injection. Pooled data from two independent experiments. *n* = 5 to 7 (two to four per experiment) per group, means ± SEM. Pairwise comparisons were performed with ordinary one-way ANOVA and corrected for multiple comparisons using the Holm-Sidak method (*P < 0.05; **P < 0.01; ***P < 0.001). (**C**) Representative MR and coregistered PET/MR images acquired 180 min post-tracer injection, with a focus on the lymphatic organs. Spleens are highlighted by white lines, dLNs are highlighted by red arrows, and ndLNs are highlighted by white arrows. (**D**) Immunofluorescence (IF) microscopy of the spleens of each group. Scale bar, 10 μm.

Furthermore, we detected higher ^64^Cu-CD4-Nb1 uptake in the spleen and tumor-draining and contralateral lymph nodes of PyMT-bearing ICI-treated hCD4-KI mice when compared to untreated PyMT- and B16F10-bearing hCD4-KI mice (Fig. 3, A to C, and fig. S2, B and C). This observation was validated by ex vivo CD4 immunofluorescence microscopy of the spleen (Fig. 3D), suggesting a systemic αPD-1/α4-1BB treatment-related increase in CD4^+^ cells in the secondary lymphoid organs.

### CD4^+^ immune cell infiltration in the tumor core is correlated with sensitivity to αPD-1/α4-1BB therapy

**Fig. 4.**
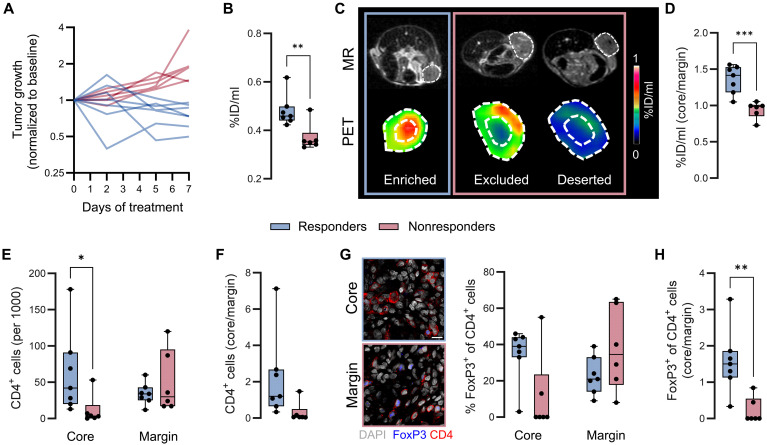
Spatial distribution of intratumoral CD4^+^ T cells determines the response to immunotherapy. (**A**) Tumor growth of PyMT hCD4-KI mice treated with αPD-1/α4-1BB antibodies (day 0 and day 3). Combined data from αPD-1/α4-1BB–treated animals of Fig. 2 (*n* = 5) and a second treatment cohort (*n* = 8). Seven mice were classified as responders (tumor volume d7/d0 < 1, blue), and six mice were classified as nonresponders (tumor volume d7/d0 > 1, red). (**B**) ^64^Cu-CD4-Nb1 PET uptake 7 days after αPD-1/α4-1BB therapy initiation in responders and nonresponders. (**C**) Representative MR (axial slice) and PET images (axial slice, tumor only) acquired 180 min after ^64^Cu-CD4-Nb1 injection. Tumors were classified as T cell–“enriched,” T cell–“excluded,” or T cell–“deserted” on the basis of the spatial distribution of ^64^Cu-CD4-Nb1 PET uptake. The tumor core and tumor margin uptake values were differentiated by a centered 50% reduced ROI (white circles). (**D**) The intratumoral CD4^+^ T cell distribution was quantified by the ratio of the tumor core and tumor margin ^64^Cu-CD4-Nb1 PET uptake. (**E**) Ex vivo quantification of CD4^+^ cells in the tumor core and the tumor margin by immunofluorescence. (**F**) Tumor core-to-margin ratios of CD4^+^ T cells. (**G**) Representative immunofluorescence images and fractions of CD4^+^FoxP3^+^ T_reg_ cells within the tumor core and tumor margin (gray: DAPI; blue: FoxP3; red: CD4). Scale bar, 10 μm. (**H**) Tumor core-to-margin ratios of CD4^+^FoxP3^+^ T_reg_ cells. Pairwise comparisons were performed with Student’s *t* test and corrected for multiple comparisons using the Holm-Sidak method (*P < 0.05; **P < 0.01; ***P < 0.001).

To further investigate whether dynamic changes in CD4^+^ T cell density within the TME provide early insights into immunotherapy efficacy, we subjected additional PyMT tumor-bearing ICI-treated hCD4-KI mice to in vivo ^64^Cu-CD4-Nb1-PET imaging. Following ICI treatment, 7 of the 13 animals were classified as responders based on inhibited tumor growth (Fig. 4A). Notably, in responsive tumors of hCD4-KI mice, ^64^Cu-CD4-Nb1 uptake was significantly higher (0.47 ± 0.03 %ID/ml) than in nonresponsive tumors (0.36 ± 0.02 %ID/ml) (Fig. 4B). In addition, we identified different uptake patterns within the TME. In responders, ^64^Cu-CD4-Nb1 predominantly accumulated in the tumor core (classified as T cell–enriched). In contrast, nonresponsive tumors showed a ubiquitously reduced ^64^Cu-CD4-Nb1 uptake (T cell–deserted) or signal accumulation predominantly at the tumor margin (T cell–excluded; Fig. 4C). To quantitatively assess these distinct spatial uptake patterns, we analyzed the tumor core and periphery separately. This was done by defining a tumor region of interest (ROI) reduced by 50% for the core, with the corresponding peripheral region encompassing the remaining area (Fig. 4C). The calculation of the core-to-periphery tracer uptake ratio determined for each tumor enabled us to differentiate between responders and nonresponders on an individual basis (Fig. 4D). These findings are in line with the results of quantitative ex vivo immunohistochemical analysis, which revealed higher CD4^+^ cell densities in the tumor core of therapy-responsive mice and higher CD4^+^ cell densities in the tumor periphery of two nonresponsive hCD4-KI mice classified with immune excluded PET uptake patterns (Fig. 4, E and F). Noteworthy, there was no correlation between therapy outcome and ^64^Cu-CD4-Nb1 uptake in the secondary lymphatic organs (fig. S3, D and E).

Unexpectedly, we detected greater fractions of FoxP3^+^ T_reg_ cells in both the tumor core of responding animals and the tumor periphery of nonresponsive animals (Fig. 4, G and H). Thus, at least in this animal model, we found that the intratumoral distribution of T_reg_ cells was associated with the CD4^+^ cell densities and no individual indicator of therapy resistance, highlighting the ability of our PET approach to capture CD4^+^ cell–mediated immune responses based on the spatial localization during treatment.

### CD4-directed PET imaging to guide treatment regimens in MC38 tumor-bearing mice

**Fig. 5.**
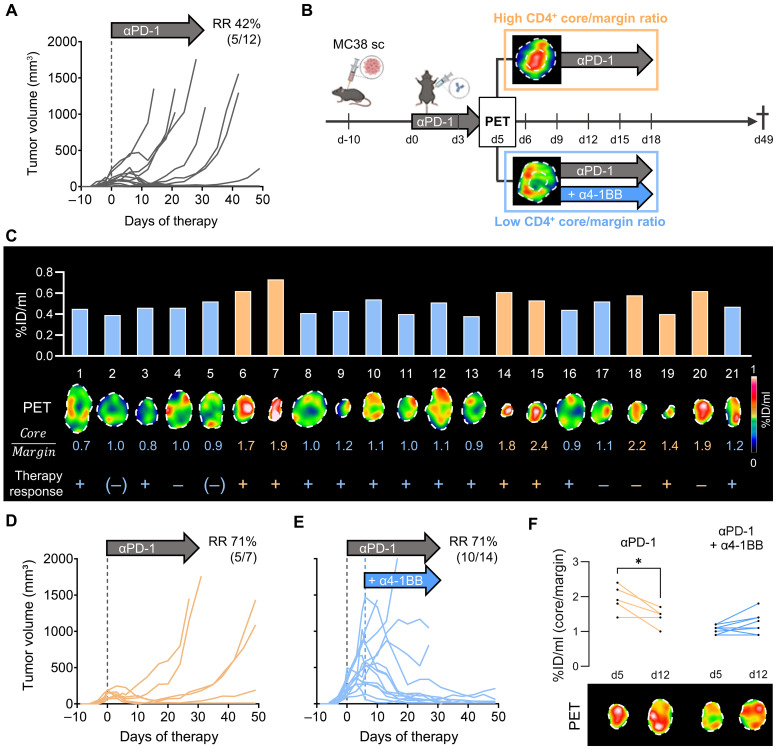
PET-guided therapy adaptation in MC38 tumor-bearing animals. (**A**) Tumor growth of αPD-1–treated mice and the related response rate (RR). MC38 tumor cells were subcutaneously (sc) implanted into C57BL/6 hCD4-KI mice 10 days before the initiation of αPD-1 mAb therapy. Antibodies were applied every 3 days for a total of seven injections. *n* = 11, data from one experiment. (**B**) Schematic illustration of the treatment adaptation based on the ^64^Cu-CD4-Nb1 PET uptake 5 days post–αPD-1 therapy initiation. Additional α4-1BB mAbs were applied starting on day 6. (**C**) ^64^Cu-CD4-Nb1 PET uptake, PET image of the tumor, calculated tumor core-to-margin ratio, and therapy response per individual mouse. Animals with an increased tumor core-to-margin ratio (>1.3) continued αPD-1 therapy (orange). αPD-1 therapy was combined with α4-1BB mAbs when the core-to-margin ratio was less than <1.3 (blue). *n* = 21, combined data from two independent experiments (*n* = 8 to 13 per experiment). (**D**) Tumor growth and related response rates of αPD-1–treated mice. (**E**) Tumor growth and related response rates of αPD-1 and subsequently added α4-1BB treatment group. (**F**) ^64^Cu-CD4-Nb1 PET core-to-margin ratio and representative PET images 5 and 12 days post-therapy initiation of mice administered either with αPD-1 monotherapy or the sequential combination of αPD-1/α4-1BB therapy. *n* = 13, data from one experiment. Pairwise comparisons were performed with Student’s *t* test and corrected for multiple comparisons using the Holm-Sidak method (*P < 0.05).

On the basis of ex vivo studies in αPD-1–treated WT animals showing a huge variety of 7.5 to 20.3% CD4^+^ T cells within the MC38 tumor infiltrate (fig. S4A), we aimed to investigate whether on-treatment CD4-PET imaging can be used to identify early therapy resistance, thus enabling timely therapy adaptation. To achieve heterogeneous therapeutic responses in a preclinical model similar to those observed in patients with cancer, we administered an ineffective dose αPD-1 treatment to MC38 colon adenocarcinoma-bearing hCD4-KI mice (*42*). Here, single αPD-1 ICI therapy resulted in a response rate of 42% (5 of 12 animals) (Fig. 5A). In a second group of animals, we applied ^64^Cu-CD4-Nb1-PET imaging 5 days after onset of αPD-1 treatment for early assessment of potential therapy resistance (Fig. 5B). Here, 33% of mice (7 of 21 animals) showed an increased ^64^Cu-CD4-Nb1 uptake in the tumor core (Fig. 5C). These mice were considered as potential responders and continued to receive αPD-1 monotherapy. Notably, all seven mice with continued αPD-1 monotherapy presented delayed tumor growth (Fig. 5D). Of these, five mice demonstrated a clear therapy response (Fig. 5D). In contrast, the animals that exhibited either enhanced tracer uptake patterns in the tumor periphery or no increased tracer uptake within the whole tumor at day 5 received α4-1BB monoclonal antibodies (mAbs) in addition to the αPD-1 antibodies from day 6 onward. This treatment was administered to overcome suspected CD4^+^ cell–mediated treatment failure. Here, another 10 of the 14 mice receiving subsequent combined αPD-1/α4-1BB therapy exhibited regressing MC38 tumors (Fig. 5E). In conclusion, we were able to increase the response rates of MC38 tumor-bearing mice from 36% in the αPD-1 monotherapy group to 71% when early CD4-PET–guided therapy adaptation was applied.

Furthermore, in the second cohort of 13 animals, we conducted additional follow-up CD4-PET imaging 12 days after therapy initiation (6 days after α4-1BB treatment). Here, we observed a significant decrease in the tumor core/margin ratio between day 5 and day 12 within the αPD-1 monotherapy group and in one animal that did not respond to combined αPD-1/α4-1BB ICI (Fig. 5F). Most animals of the combined αPD-1/α4-1BB ICI group demonstrated a stable or increased tumor core/margin ratio at the later imaging time point, suggesting that α4-1BB therapy increased the influx of CD4^+^ T cells into the TME (Fig. 5F).

### Low intratumoral CD4^+^ T cell densities are associated with early relapse after neoadjuvant αPD-L1 therapy in patients with NSCLC

**Fig. 6.**
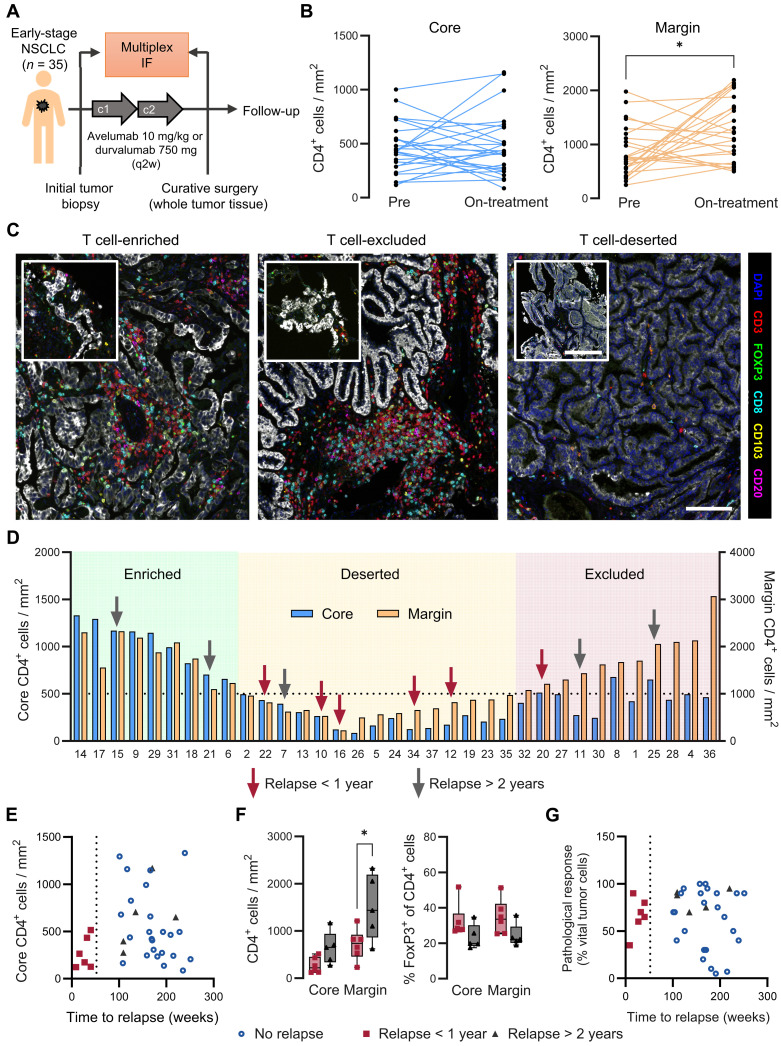
Ex vivo multiplex immunofluorescence of whole tumor samples from patients with NSCLC treated with neoadjuvant PD-L1 ICI. (**A**) Schematic illustration of the clinical study. (**B**) Quantification of CD3^+^CD8^−^ T cell densities (considered as CD4^+^ T cells) within the tumor core (left) and tumor margin (right) from pretreatment and on-treatment tissue. *n* = 35, combined data from two independent clinical studies. (**C**) Multiplex immunofluorescence microscopy images of the indicated seven-marker antibody panel. Tumor biopsy samples before treatment (upper left image) and corresponding posttreatment whole tumor samples from three representative cases are displayed. Scale bars, 100 μm. (**D**) Classification of T cell–enriched, T cell–deserted, and T cell–excluded tumors on-treatment on the basis of the CD4 core and margin quantification. Red arrows indicate that patients relapsed within 1 year after surgery (*n* = 6). Gray arrows indicate that patients relapsed after more than 2 years after surgery (*n* = 5). (**E**) Correlation between the time to relapse after surgery and the number of CD4^+^ cells within the core of the resected tumors. (**F**) Tumor core and margin densities of CD4^+^ cells (left) and FoxP3^+^ T_reg_ fraction of CD4^+^ cells (right) of patients relapsed within 1 year and >2 years after surgery. (**G**) Correlation of time to relapse after surgery and pathological response based on the fraction of vital cells. Pairwise comparisons were performed with Student’s *t* test and corrected for multiple comparisons using the Holm-Sidak method (*P < 0.05).

Last, we aimed to determine the extent to which the differential distribution patterns of tumor-infiltrating CD4^+^ cells could serve as predictors of therapy efficacy in a clinical setting. For this purpose, we analyzed the immune cell infiltration in 35 tumor biopsies (pretreatment) and subsequent whole tumor samples (on-treatment) from patients with NSCLC enrolled in two prospective clinical trials (NCT03853187 and NCT03514719) and treated with two cycles of αPD-L1 mAbs (durvalumab or avelumab) in a neoadjuvant setting (Fig. 6A). Because the multiplex immunofluorescence panel lacked CD4, CD3^+^CD8^−^ cells were classified as CD4^+^ T cells after confirming that the densities of CD4^−^CD8^−^ cells were <10% of all T cells within the TME (fig. S4, B and C). As expected from the relatively low response rates in patients with NSCLC, CD4^+^ T cells were significantly enriched in the tumor margin, whereas only a few patients presented increased CD4^+^ T cell accumulation within the tumor core between baseline and after αPD-L1 treatment (Fig. 6B). On the basis of the on-treatment CD4^+^ T cell densities at the tumor core and margin, we differentiated T cell–enriched (*n* = 9), T cell–excluded (*n* = 11), and T cell–deserted tumors (*n* = 15) (Fig. 6, C and D). Notably, five of the six patients who relapsed within 1 year after surgery were classified as having T cell–deserted tumors on-treatment, and all patients with NSCLC exhibited low CD4^+^ T cell densities within the tumor core (Fig. 6E). Patients with NSCLC who experienced a later relapse (after >2 years) revealed an increased CD4^+^ T cell density in both the tumor core (nonsignificant) and the tumor margin compared to patients with early relapse (<1 year) (Fig. 6F). In line with our preclinical data, we did not detect relevant changes in the fraction of FoxP3^+^ T_reg_ cells between the relapse groups and the intratumoral location (Fig. 6F). These observations suggest that spatial CD4^+^ T cell distribution is a clinically relevant biomarker for primary (low core CD4) and secondary (high margin CD4) resistance to αPD-(L)1 therapy. In contrast, the pathological response, which is based on the fraction of residual vital tumor cells upon treatment and considered as the gold standard for the evaluation of therapy responsiveness in neoadjuvant regimens, was not correlated with patient relapse (Fig. 6G).

## DISCUSSION

Starting with the approval of the CTLA-4–targeting ICI ipilimumab for metastatic melanoma in 2011, the subsequent decade of immunotherapy has introduced several antibody-based, cell-based, and vaccine-based treatment options for patients with cancer, with many more options expected in the next 5 to 10 years (*43*). However, all these approaches suffer from limited response rates and a lack of reliable biomarkers to predict therapeutic efficacy (*44*). Tumor-derived parameters such as immunohistochemical assessed PD-(L)1 expression or tumor mutational burden (*45*–*48*) and blood-based biomarkers (*49*–*51*) have been identified as promising indicators for predicting therapeutic efficacy. However, even artificial intelligence–based multimodal integration of these different parameters has demonstrated very limited predictive value in larger patient cohorts (*52*, *53*).

On the basis of the hypothesis that immune responses and resistance mechanisms become visible only after treatment has been initiated, we introduced a noninvasive imaging approach for the early monitoring of immunotherapy. In contrast to cancer cell–specific targets (such as PSMA, HER-2, and SSTR), immune cell imaging faces the challenge of limited target-expressing cell densities, necessitating tracers with exceptional sensitivity and spatial resolution to achieve accurate detection. Recently, Nbs have been introduced in the diagnostic field for their unique structural and functional properties, including high stability and binding affinity, and low molecular weight. In a previous study (*38*), we developed and extensively validated different CD4-targeting Nbs and selected our lead candidate, which exhibited the ability to quantitatively assess CD4^+^ cells within the TME and lymphatic tissues. The advantageous biological properties of Nbs, as well as the high spatial resolution of ^64^Cu (*54*, *55*), allowed us to differentiate clinically relevant variations in CD4^+^ cell densities and even intratumoral distribution patterns of mouse tumors with diameters below 5 mm. Our hCD4-KI versus WT mouse comparisons demonstrated that specific uptake is not affected by varying levels of CD4 antigen in the system, which occurs in the context of chemotherapeutics or CD4^+^-depleting therapies. This characteristic is unique compared with other published CD4-targeting PET tracers with human or murine specificity (*23*–*28*, *56*, *57*). Notably, fluorescence-based imaging approaches have been developed to track CD4^+^ T helper and other immune cells with high sensitivity (*58*, *59*). However, the limited tissue penetration depth and the semiquantitative nature of the signals largely restrict the clinical use of optical imaging to certain endoscopic or cutaneous applications.

CD4^+^ cells comprise a diverse group of T helper cells, T_reg_ cells, and T effector cells, and as such, their role as predictive biomarkers remains a topic of ongoing and controversial debate (*1*, *2*, *32*–*36*, *60*–*64*). Moreover, the CD4 antigen is also expressed to a lesser extent on other immune cells, including subsets of natural killer cells, monocytes, and macrophages (*65*). In contrast to inducible immune cell targets (*66*, *67*), CD4 expression exhibits only minor variations following antigenic activation (*68*), virus infection (*69*) or epigenetic regulation during thymic differentiation (*70*), thereby enabling accurate quantification of CD4^+^ cells by PET.

Through ^64^Cu-CD4-Nb1 PET imaging, we could spatially localize CD4^+^ cells within the TME of both immunologically “cold” B16F10 melanomas and “hot” PyMT breast tumors, discerning minimal variations in CD4^+^ cell densities in the TME (*23*, *71*). Notably, we observed different CD4^+^ cell infiltration patterns in PyMT tumors with ineffectively αPD1/α4-1BB–treated animals showing infiltration predominantly at the tumor periphery, whereas effectively treated animals exhibited increased infiltration within the tumor core.

Consistent with our findings of increased CD4-derived PET uptake in lymph nodes and spleens, Kim *et al.* similarly observed the accumulation of CD4^+^ and CD8^+^ cells in αPD-1/α4-1BB–treated C57BL/6 mice. The authors further reported pronounced lymph node swelling and interferon-γ–driven macrophage recruitment following repeated α4-1BB administration, which they associated with impaired CD8^+^ T cell function and the development of adverse events (*72*).

T_reg_ cells have been reported to form physical, metabolic, and trafficking barriers that limit the entry of effector T cells into the tumor core (*73*). Thus, we hypothesized that the CD4-derived tracer signal from tumor margins in nonresponding animals were due to increased accumulation of T_reg_ cells, which suppress the therapeutic response and immune cell infiltration into the tumor. Ex vivo immunofluorescence of mouse and NSCLC patient samples confirmed the correlating accumulation of both CD4^+^ helper cells and T_reg_ cells in the tumor periphery and in the tumor core, suggesting that immune effector function induces regulatory immune responses irrespective of therapy response or resistance.

To substantiate the value of CD4-directed PET imaging as a decision-making approach, we applied additional α4-1BB mAbs, which have been shown to deplete T_reg_ cells (*74*), to standard-of-care αPD-1–treated MC38 tumor-bearing animals, which presented increased ^64^Cu-CD4-Nb1 PET tracer uptake in the tumor periphery. Most animals were correctly selected for therapy response, and combined treatment was successful even in mice with larger tumors. In tumor samples from patients with NSCLC treated with ICIs, high CD4^+^ cell densities in the tumor cores correlated with favorable clinical outcomes, highlighting the clinical utility of our noninvasive PET imaging approach.

With respect to clinical application, ^64^Cu is a valuable radionuclide, combining preferable decay characteristics (radiation exposure) compared to frequently used long-living isotopes such as ^89^Zr, with the advantages of centralized tracer production and continent-wide overnight shipping capabilities (*54*, *55*). Nevertheless, combining Nb technology with fluorine-18 (^18^F) labeling, which has recently been established for Nbs (*75*, *76*), could pave the way for multiparametric imaging approaches allowing noninvasive 1-day visualization and localization of multiple immune cell populations, thereby providing a comprehensive assessment of a patient’s immune status without relying on invasive techniques such as biopsy (*23*, *28*, *77*).

Various metabolic factors such as hypoxia (*78*), immunological components including suppressive immune cells, cytokines, and MHC-II expression (*79*), as well as tumor-intrinsic drivers such as oncogenic mutations (e.g., *KRAS*, *TP53*, and *BRAF*) and aberrant signaling pathways (*80*), collectively influence the infiltration of CD4^+^ T cells. To better understand the underlying mechanisms of our findings, it will be crucial to colocalize the PET-based macroscopic distribution patterns of CD4^+^ T cells with spatial transcriptomics and immunogenomics.

In conclusion, analysis of CD4-directed PET tracer uptake patterns allowed us to identify distinct immune features within the TME that are associated with sensitivity to ICIs and have the potential to guide the development and optimization of combinatorial immunotherapy strategies. Moreover, the spatially resolved ^64^Cu-CD4-Nb1-PET data of the TME enables us to forecast treatment resistance, which was further validated through immunofluorescence analysis in both mouse and human NSCLC tumor tissues. Thus, Nb-based PET imaging of CD4^+^ cells provides mechanistic insights into the therapeutic actions of immunotherapies and underlying resistance mechanisms, extending beyond the mere monitoring of therapeutic responses. This approach holds great potential for guiding combinatorial immunotherapy strategies in patients with cancer.

## MATERIALS AND METHODS

### Nb production

CD4-Nb1 and GFP-Nb were either produced and labeled as previously described (*38*), or adapted versions that allow for site-directed chemical conjugation (*81*) were produced using the ExpiCHO Expression System according to the manufacturer’s protocols (Thermo Fisher Scientific, Germany). The latter Nbs were isolated from the cell-free culture supernatant by a combination of protein affinity chromatography, anion exchange chromatography, and cation exchange chromatography. Nbs were reacted with *p*-NCS-benzyl-NODA-GA (Chematech, France) to produce Nb-NODAGA conjugates. The integrity and high purity of the Nbs and Nb-NODAGA conjugates were confirmed via liquid chromatography–mass spectrometry (LC-MS) and SDS–polyacrylamide gel electrophoresis. The comparability of the Nb-NODAGA conjugates from the two production systems was confirmed by highly similar target-binding affinities using biolayer interferometry (Octet RED96e system, Sartorius).

### Radiolabeling

[^64^Cu] CuCl_2_ [150 megabecquerel (MBq) in 0.1 M HCl] was neutralized by addition of 1.5 volumes of 0.5 M ammonium acetate solution (pH 6), resulting in a pH of 5.5. To this solution, 50 μg of the conjugate was added and incubated at 42°C for 60 min. Then, 1 μl of 20% diethylenetriamine pentaacetic acid solution was added to quench the labeling reaction. Complete incorporation of the radioisotope was confirmed after each radiosynthesis by thin layer chromatography [iTLC-SA; Agilent Technologies; mobile phase: 0.1 M citric acid (pH 5)] and high-performance size exclusion chromatography [HPSEC; BioSep SEC-s2000, 300 mm by 7.8 mm, Phenomenex; mobile phase: Dulbecco’s Balanced Salt Solution (DPBS) with 0.5 mM EDTA]. All radiolabeled preparations used for in vivo PET imaging had radiochemical purities of ≥90% (iTLC and HPSEC).

### Bead assay

A total of 20 μl of Ni-NTA beads (nickel–nitrilotriacetic acid; HisPur Ni-NTA magnetic beads; Thermo Fisher Scientific) was washed with 380 μl of phosphate-buffered saline (PBS) containing 0.05% Tween 20 (PBS-T), vortexed for 5 s, and placed on a magnetic rack (12321D; DynaMag-2; Thermo Fisher Scientific) for 30 to 45 s to isolate the magnetic beads. The His-tagged hCD4 antigen (Thermo Fisher Scientific) was resuspended as per the manufacturer’s instructions to achieve a concentration of 0.1 mg/ml. The washed beads were resuspended in 190 μl of PBS-T, incubated with 1 μg of His-tagged hCD4 antigen for 15 min on a rotating mixer at room temperature (RT), and then washed with PBS-T. A large excess (1 μg) of the unlabeled ligand was added to the antigen-coated beads in the blocking arm and incubated for 15 min on a rotating mixer at RT. Afterward, 1 ng of ^64^Cu-CD4-Nb1 was incubated with antigen-coated beads for 30 min on a rotating mixer at RT. The beads were isolated using a magnet, and half of the supernatant (SN) was transferred to a γ-counter tube. The solutions of beads + SN (BS) and SN only (S) were measured by γ-counting (Wallac 1480 WIZARD 3” Gamma Counter; PerkinElmer, Waltham, MA, USA), and the immunoreactive fraction was calculated as (BS − S)/(BS + S).

### Tumor cells

hCD4^−^ diffuse histiocytic lymphoma (DHL) cell lines were purchased from the German Collection of Microorganisms and Cell Cultures (DSMZ, Braunschweig, Germany) and cultured in RPMI 1640 medium supplemented with 10% fetal calf serum (FCS) and 1% penicillin/streptomycin (P/S). The B16F10 murine melanoma cell line was purchased from the American Type Culture Collection (ATCC) and cultured in Dulbecco’s modified Eagle’s medium (DMEM) supplemented with 10% FCS and 1% P/S. The S2WTP3 (PyMT) triple-negative breast cancer cell line was kindly provided by A. Moeller (Queensland University, Australia) and cultured in DMEM supplemented with 10% FCS, 1% P/S, and 1% pyruvate (Sigma-Aldrich). The MC38 murine colon adenocarcinoma cell line was purchased from Kerafast and cultured in DMEM supplemented with 10% FCS, 1% P/S, and 1% Hepes.

### In vitro binding assays

The binding of ^64^Cu-CD4-Nb1 or ^64^Cu-GBP-Nb to freshly isolated hCD4^+^ T cells and hCD4^−^ SU-DHL-4 cells was assessed in vitro. A total of 1 × 10^6^ cells was incubated with 2 ng of ^64^Cu-CD4-Nb1 or ^64^Cu-GBP-Nb for 90 min at 37°C on a shaker. The cells were washed twice with PBS/1% FCS and resuspended in 200 μl. The radioactivity was measured by γ-counting.

### Animals

All experiments were performed according to the animal use and care protocols (R 04/22 G, R 08/21 G) of the German Animal Protection Law and were approved by the local authorities (Regierungspräsidium Tübingen). Eight- to 16-week-old WT C57BL/6J (Charles River Laboratories) and hCD4-KI (C57BL/6J-*Cd4*^*tm1.1(CD4)Geno*^, Genoway, in-house breeding) mice were bred under specific pathogen–free conditions with free access to food and water ad libitum.

### Tumor models

A total of 0.12 × 10^6^ B16F10 tumor cells was implanted by intradermal injection into hCD4-KI mice in PBS. A total of 0.5 × 10^6^ to 0.6 × 10^6^ PyMT mammary tumor cells were orthotopically injected into the fourth mammary fat pad in PBS into C57BL/6J or hCD4-KI mice. WT or hCD4-KI mice with PyMT tumors were injected intraperitoneally with 200 μg of αPD-1 (clone: RMP1-14; Bioxcell) and 50 μg of α4-1BB (clone: 3H3; Bioxcell) mAbs 7 days prior to in vivo imaging. hCD4-KI mice with MC38 tumors were injected intraperitoneally with 100 μg of αPD-1 mAbs every 3 days for a total of seven injections. Animals with low tumor core PET tracer uptake on day 5 received five additional α4-1BB antibodies (50 μg) beginning on day 6 in combination with the αPD-1 mAbs.

### PET/MR imaging

For noninvasive in vivo imaging, 5 μg (~2 MBq/μg) ^64^Cu-CD4-Nb1 or ^64^Cu-GFP-Nb was administered intravenously via the tail vein of experimental mice. PET and magnetic resonance (MR) scans were acquired at 5 and 90 min and 3 and 6 hours post-tracer injection (longitudinal scans) or only at the 3-hour post-tracer injection time point. Anatomic images were acquired by MR on a dedicated 7T small animal MR tomograph (ClinScan; Bruker BioSpin, Ettlingen, Germany). A T2-weighted image three-dimensional space sequence [repetition time (TR): 1800 ms, echo time (TE): 4.7 ms, field of view (FoV): 76.8 mm by 34.8 mm by 22.8 × mm, matrix: 256 × 116 × 76] was acquired in ~9 min. The animals were kept under 1.5% isoflurane (100% oxygen) anesthesia, and the breathing frequency was monitored with a control unit (SA Instruments, Stony Brook, NY, USA). Static PET images (600 s) were acquired with dedicated Inveon small animal PET scanners (Siemens Preclinical Solutions, Knoxville, TN, USA).

### Image analysis

PET images from list mode were reconstructed using three-dimensional ordered subset expectation maximization (OSEM-3D) and coregistered to the anatomical T2 MR images using Inveon Research Workplace (Siemens Preclinical Solutions). The volumes of interest (VOIs) of the organs of interest were created based on the anatomical MR images. The uptake values of the respective organs were calculated from the mean Bq/ml, corrected for radioactive decay and normalized to the injected activity, and presented as percentage injected dose per milliliter (%ID/ml).

Spatial distribution analyses were performed by defining tumor margin and tumor core ROIs as 50% of the tumor diameter in a centered cross section of the PET-acquired image using open-source ImageJ software. PET signal intensity was quantified as the mean intensity of the tumor margin and core.

### Ex vivo biodistribution

The experimental mice were euthanized by cervical dislocation under deep anesthesia after the final imaging time point. Organs were harvested, and radioactivity was measured by γ-counting. For quantification, standardized aliquots of the injected tracer were added to the measurement. The values for each organ are expressed as the percentage of the overall injected dose per gram (%ID/g), corrected for radioactive decay and normalized to the injected activity.

### Immunohistochemistry of PyMT tumors

After the organs were γ-counted, the tumors, spleens, and draining and contralateral nondraining lymph nodes were fixed in formalin and embedded in paraffin. For histology, 3- to 5-μm-thick sections from paraffin-embedded tissues were cut and stained with hematoxylin and eosin (H&E). Immunohistochemistry (IHC) was performed on an automated immunostainer (Ventana Medical Systems Inc.) according to the manufacturer’s protocols for open procedures, with slight modifications. All the slides were stained with antibodies against anti-human CD4 (SP35, Zytomed). Appropriate positive and negative controls were used to confirm the adequacy of the staining. All the samples were scanned with a Ventana DP200 (Roche, Basel, Switzerland) and processed with the Image Viewer MFC application. The final image preparation was performed with Adobe Photoshop CS6. The number of CD4^+^ T cells was counted in at least 10 high-power fields (HPFs). The data are presented as the number of CD4^+^ T cells per field.

### Immunofluorescence microscopy of PyMT tumors and spleens

Fresh-frozen 5-μm cryosections fixed with iodate-lysine-paraformaldehyde or sections from paraffin-embedded tissues were blocked with donkey serum and incubated with rabbit anti-FoxP3 (polyclonal, Novus Biologicals, USA) or rabbit anti-CD3 (DCM-39, DCS, Germany) and goat anti-CD4 (polyclonal, R&D Systems, USA). Bound mAbs were visualized with Alexa Fluor 647–Donkey anti-rabbit-F(ab′)2 (H+L, Dianova, USA) and Cy3-Donkey anti-goat-F(ab′)2 (H+L, Dianova). For nuclear staining, we used 4′,6-diamidino-2-phenylindole (DAPI) (Sigma-Aldrich). Immunofluorescence images were acquired with an LSM 800 confocal laser scanning microscope (Carl Zeiss) and processed with ZEN Blue software, version 2.6. The analysis of cell nuclei was performed using Zeiss Zen software, whereas the number of CD4^+^, FoxP3^+^, and CD4^+^FoxP3^+^ cells was counted manually. For each section, the sum of a minimum of six different areas—three intratumoral and three marginal—was normalized to the automated counted cell number based on the DAPI-derived nuclei.

### Multiplex immunofluorescence of human lung cancer samples

Baseline biopsy and on-treatment resected tumor samples from patients with early-stage NSCLC enrolled in either of two prospective clinical trials, NCT03514719 or NCT03853187, were subjected to multiplex immunofluorescence. In these trials, patients received two courses of neoadjuvant αPD-L1 antibodies (10 mg/kg avelumab) or 750 mg durvalumab at a 2-week interval, followed by surgical resection. A formalin-fixed, paraffin-embedded material was cut into 4-μm-thick sections and mounted on Superfrost PLUS slides (AR9222, Leica Biosystems). Automated mIHC staining using the Opal 7-Color Automation IHC Kit, Opal480, and Opal780 (NEL821001KT, FP1500001KT, and FP1501001KT, Akoya Biosciences) was performed on the Leica Bond system (BOND-Rx Fully Automated IHC and ISH, Leica Biosystems), as described previously (*82*–*84*). This staining identified CD8^+^ T cells (CD3^+^CD8^+^), CD4^+^ T cells (CD3^+^CD8^−^), T_reg_ cells (CD3^+^FoxP3^+^), tissue-resident T cells (CD3^+^CD103^+^CD8^+/−^), and B cells (CD20^+^) (*83*). To quantify lymphocytes in different tumor regions, pretreatment biopsy and posttreatment resection materials were segmented into tumor cores and invasive margins via Qupath software (*84*, *85*). These regions were confirmed by a certified pathologist. The invasive margin was defined as the portion of the TME where tumor cells interface with the surrounding tissue, and the maximum distance of the invasive margin was set at 100 μm surrounding the tumor core (*84*).

### Statistical analysis

All the data were analyzed using GraphPad Prism, version 9 or later (GraphPad Software Inc., San Diego, CA, USA). The values are expressed as the arithmetic means ± SD for single experiments or as the arithmetic means ± SEM for combined experiments. For statistical analyses, unpaired/paired *t* tests were applied for pairwise comparisons. Ordinary one-way analysis of variance (ANOVA) or two-way ANOVA was used for multiple group comparisons and was corrected for multiple comparisons using the Sidak post hoc test. Adjusted *P* values less than 0.05 were considered significant, and significance levels are indicated as follows: *P ≤ 0.05, **P ≤ 0.01, and ***P ≤ 0.001.
