# Supplementary material for: PET-based immunomapping of intratumoral CD4+ cells to monitor acquired resistance to checkpoint inhibitors
Source: Sci Adv. 2025 Jun 25;11(26):eadw1924. doi: 10.1126/sciadv.adw1924 (PMC12190014; doi:10.1126/sciadv.adw1924)
Supplement: Supplementary file 1 — Figs. S1 to S4 [file sciadv.adw1924_sm.pdf]

Science Advances
AAAS

Supplementary Materials for

PET-based immunomapping of intratumoral CD4^{+} cells to monitor acquired resistance to checkpoint inhibitors

Stefania Pezzana *et al.*

Corresponding author: Dominik Sonanini, dominik.sonanini@med.uni-tuebingen.de

Sci. Adv. **11**, eadw1924 (2025)
DOI: 10.1126/sciadv.adw1924

This PDF file includes:

Figs. S1 to S4

SUPPLEMENTARY MATERIAL

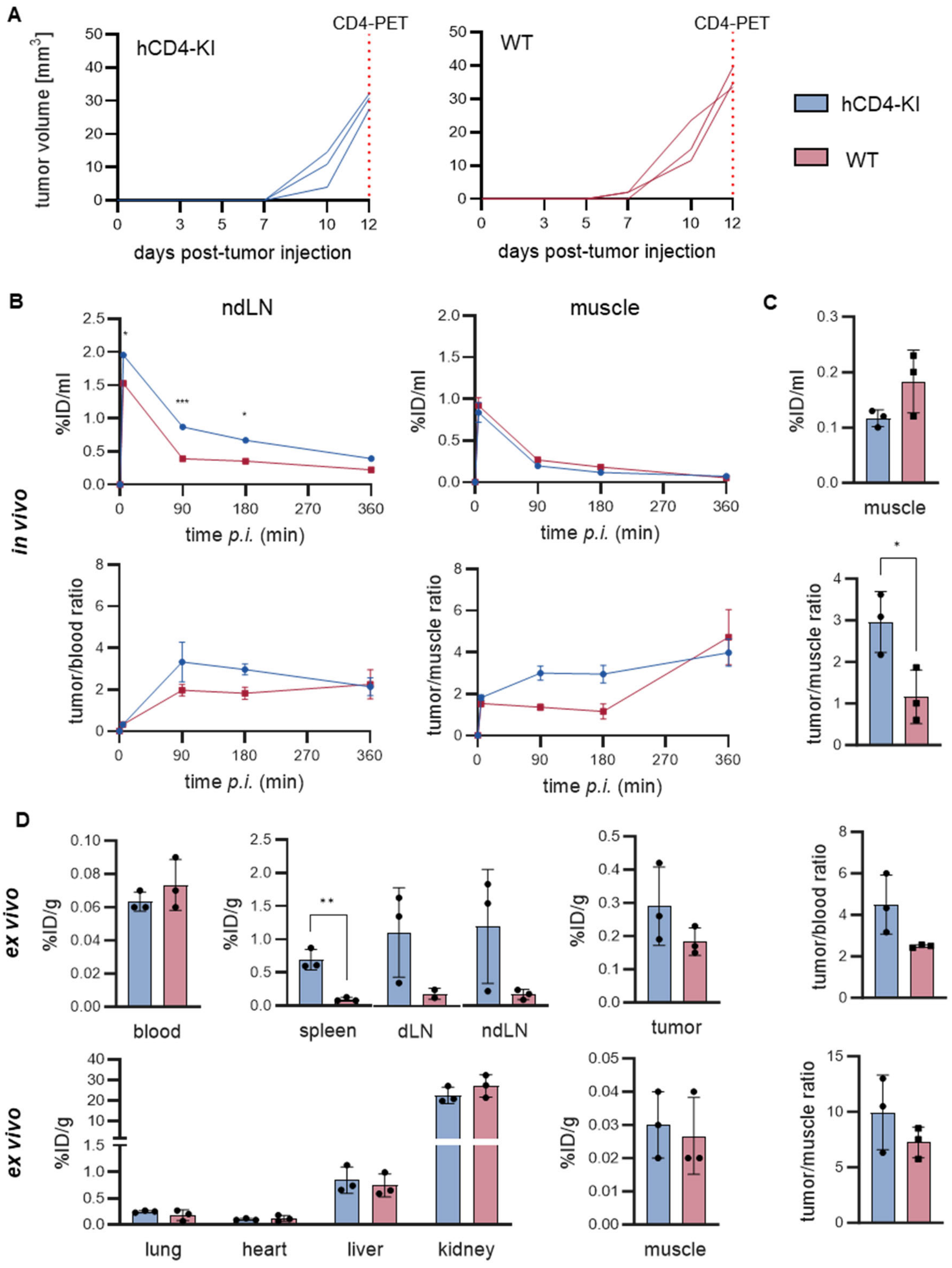

Fig. S1. Specific binding and biodistribution dynamics of ^{64}Cu-CD4-Nb1. (A) PyMT tumor growth in hCD4-KI and WT mice. red line: ^{64}Cu-CD4-Nb1 tracer injection. (**B**) ^{64}Cu-CD4-Nb1 PET uptake dynamics 5 to 360 min post-tracer injection (*p.i.*) in non-draining lymph nodes (ndLN), muscle, tumor-to-blood and tumor-to-muscle ratio (n=3 per group). Data are given as mean percent of injected dose per ml (%ID/ml). (**C**) ^{64}Cu-CD4-Nb1 PET uptake quantification 80 minutes post-tracer injection in muscle and tumor-to-muscle ratio. (**D**) *Ex vivo* ^{64}Cu-CD4-Nb1 uptake quantification in blood, spleen, draining lymph node (dLN), ndLN, tumor, lung, heart, liver, kidney, and tumor-to-blood and tumor-to-muscle ratios. Pairwise comparisons were performed with Student's t-test and corrected for multiple comparisons using the Holm-Sidak method (*$p < 0.05$, **$p < 0.01$, ***$p<0.001$).

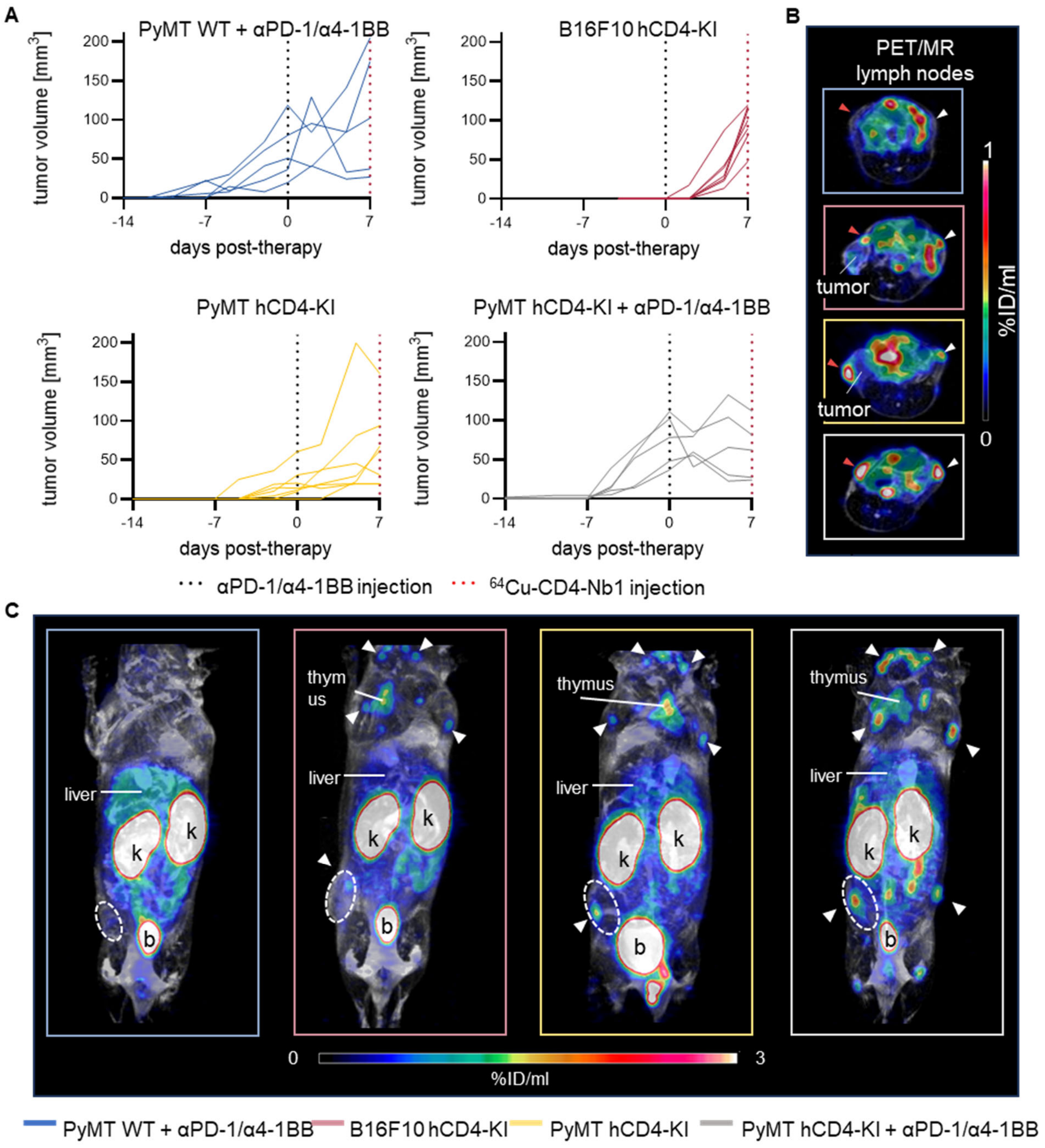

Fig. S2. ^{64}Cu-hCD4-Nb1 PET to quantify varying intratumoral CD4^{+} cell densities. (A) PyMT and B16F10 tumor growth in hCD4-KI and WT mice. Black dotted line: αPD-1/α4-1BB treatment injection; red line: ^{64}Cu-CD4-Nb1 tracer injection. (**B**) Representative axial PET/MR images at 180 minutes post ^{64}Cu-CD4-Nb1 tracer injection. Red triangle: draining lymph node; white triangle: non-draining lymph node. (**C**) Representative maximum intensity projection (MIPs) PET images overlaid with anatomical MRI, acquired 180 min after ^{64}Cu-CD4-Nb1 injection in human CD4 knock-in (hCD4-KI) or wildtype (WT) mice with orthotopic PyMT triple negative breast cancer tumors and B16F10 melanoma. Tumors are outlined with a white circle, lymph nodes are highlighted by white arrows, k: kidney; b: bladder.

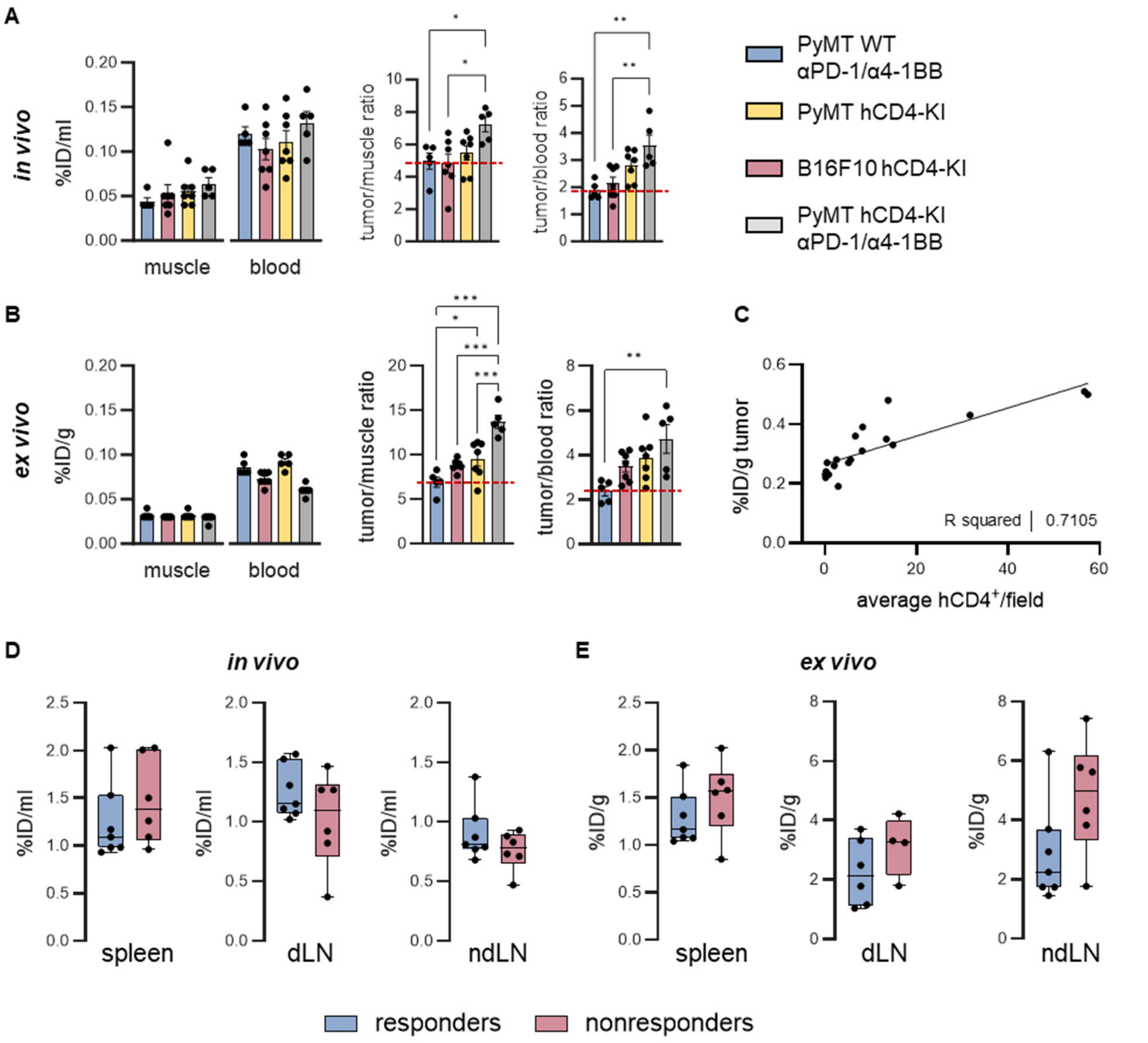

Fig. S3. ^{64}Cu-hCD4-Nb1 PET and *ex vivo* correlation CD4^{+} cell densities. (**A**) Quantification of *in vivo* and (**B**) *ex vivo* ^{64}Cu-CD4-Nb1 uptake at 180 minutes post-tracer injection and the respective tumor-to-muscle and tumor-to-blood ratios. Red horizontal lines represent background levels based on the WT group. (**C**) Correlation of *ex vivo* tumor uptake with IHC mean of hCD4^{+} cell per field of view. (**D**) *In vivo* ^{64}Cu-CD4-Nb1 PET uptake and (**E**) *ex vivo* biodistribution of spleen, draining lymph nodes (dLN) and non-draining lymph nodes (ndLN) of PyMT hCD4-KI mice treated for 7 days with αPD-1/α4-1BB antibodies (day 0 and day 3). Combined data from αPD-1/α4-1BB treated animals of Fig. 2 (n = 5) and a second treatment cohort (n = 8). Seven mice were classified as responders (tumor volume d7/d0 <1, blue), and 6 mice were classified as nonresponders (tumor volume d7/d0 >1, red). Pairwise comparisons were performed with one-way ANOVA and corrected for multiple comparisons using the Holm-Sidak method (*$p < 0.05$, **$p < 0.01$, ***$p < 0.001$).

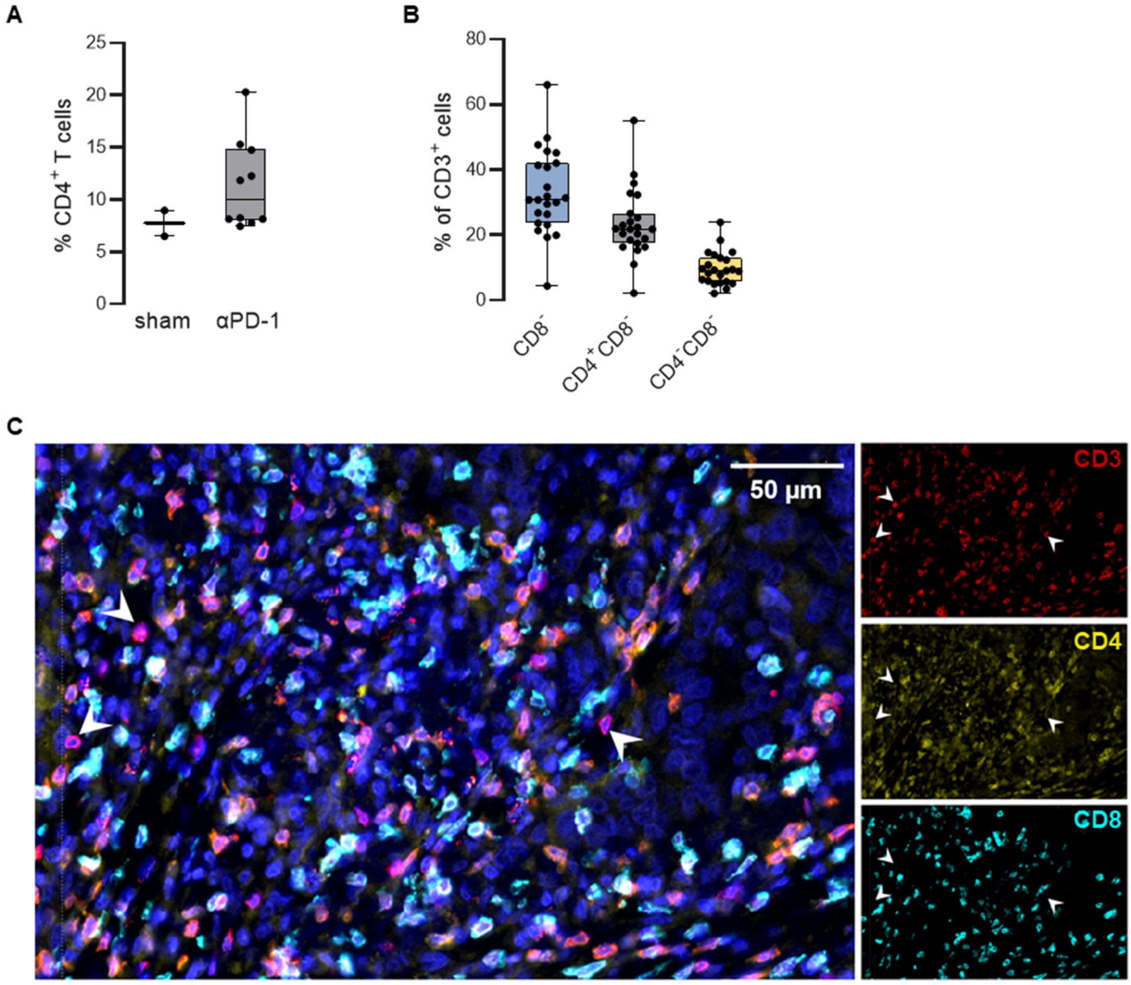

Fig. S4. *Ex vivo* validation of CD4$^+$ T cell infiltration in αPD-1-treated MC38 tumor-bearing animals and of CD4$^+$ T cell populations in human NSCLC tissue. (**A**) Mass cytometry of CD3+CD4+ T cell infiltration (day 3) of C57BL/6 mice with MC38 tumors after single sham or αPD-1 treatment (100 µg per mouse). Data was extracted from a 26-plex antibody panel with ^{152}Sm-CD3e (clone 145-2C11, Standard Biotools) and ^{172}Yb-CD4 (RM4-5, Standard Biotools) acquired in a Helios CyTOF System (Standard Biotools) and analyzed using FlowJo software (BD Bisociences). (**B**) Comparison of different CD4$^+$ T cell gating strategies and (**C**) representative multiplex immunofluorescence microscopy from 23 human NSCLC tissues. Images were obtained from a tissue microarray containing tumor regions of interest and analyzed using the PhenoCycler®-Fusion 2.0 system (Akoya Biosciences). A multiplex panel of 39 purified antibodies labeled with Cy3 or Cy5 was employed, and fluorescence signals from DAPI, CD3 (clone MRQ-39, CellMarque), CD4 (clone EPR6855, abcam), and CD8 (clone C8/144B, Santa Cruz Biotechnology) were depicted for cell segmentation via DeepCell (v0.12.6) and the Enable Medicine platform. White arrows indicate CD3$^+$CD4$^-$CD8$^-$ cells.
